# Detection of Oncogene Hotspot Mutations in Female NSCLC Tumor DNA and Cell-Free DNA

**DOI:** 10.3390/cancers16091770

**Published:** 2024-05-03

**Authors:** Ieva Drejeriene, Saulius Cicenas, Diana Stanciute, Arnoldas Krasauskas, Jurate Gruode

**Affiliations:** 1 Faculty of Medicine, Vilnius University, 03101 Vilnius, Lithuania; 2 Klaipeda University Hospital, 92288 Klaipeda, Lithuania; jurate.gruode@kul.lt; 3 National Cancer Institute, 08406 Vilnius, Lithuania; 4 Faculty of Health Science, Klaipeda University, 92294 Klaipeda, Lithuania

**Keywords:** non-small-cell lung cancer, cell-free DNA, plasma, NGS

## Abstract

**Simple Summary:**

Non-small-cell lung cancer is the most prevalent type of lung cancer, with extensively characterized mutational spectra. Several biomarkers (such as *EGFR*, *BRAF*, *KRAS* gene mutations, etc.) have emerged as predictive and prognostic markers for lung cancer. Unfortunately, the quality of the available tumor biopsy and/or cytology material is not always adequate to perform the necessary molecular testing, prompting the search for alternatives. Cell-free DNA found in plasma is emerging as a highly promising avenue or a supplementary method for assessing the efficacy of cancer treatments. In this study, 51 Lithuanian females with non-small-cell lung cancer were studied. From each woman, two samples were obtained: lung tumor and plasma. Target mutations were identified in 38 out of 51 patients in tumor tissue samples, while in plasma samples, they were identified in only 10 patients’ samples. Theoretically, cfDNA from plasma could be a superior tool for lung cancer diagnostics and the detection of early cancer stages. Therefore, further improvements in cfDNA extraction and detection methods from plasma are needed, including the development of superior detection kits and analysis tools.

**Abstract:**

Non-small-cell lung cancer (NSCLC) is the most prevalent type of lung cancer, with extensively characterized mutational spectra. Several biomarkers (such as *EGFR*, *BRAF*, *KRAS* gene mutations, etc.) have emerged as predictive and prognostic markers for NSCLC. Unfortunately, the quality of the available tumor biopsy and/or cytology material is not always adequate to perform the necessary molecular testing, prompting the search for alternatives. Cell-free DNA (cfDNA) found in plasma is emerging as a highly promising avenue or a supplementary method for assessing the efficacy of cancer treatments. This is especially valuable in instances where conventional biopsy specimens, like formalin-fixed, paraffin-embedded (FFPE), or freshly frozen tumor tissues prove inadequate for conducting molecular pathology analyses subsequent to the initial diagnostic procedures. By leveraging cfDNA from plasma, clinicians gain an additional tool to gauge the effectiveness of cancer therapies, thereby enhancing their ability to optimize tailored treatment strategies. In this study, 51 Lithuanian females with NSCLC were analyzed, with adenocarcinoma being the predominant pathology diagnosis in 40 cases (78%). Target mutations were identified in 38 out of 51 patients (74.5%) in tumor tissue samples, while in plasma samples, they were identified in only 10 patients’ samples (19.6%). Even though we did not have enough voluminous plasma samples in our study, gene mutations were detected in plasma from ten women, three of whom were diagnosed with early stages of lung cancer (stages I and II). For these patients, the following mutations were detected: deletion in exon 19 of the *EGFR* gene and single nucleotide polymorphisms in the *TP53* and *MET* genes. All other women were diagnosed with stages III or IV of lung cancer. This indicates that the later stages of cancer contribute more cfDNA in plasma, making extraction less complicated.

## 1. Introduction

Molecularly targeted therapies against driver mutations in patients with lung cancer, particularly non-small-cell lung cancer (NSCLC), are already improving patients’ survival over traditional chemotherapy [1]. Consequently, molecular genetic testing should be routinely applied in clinical practice. Currently, the successful use of immune checkpoint inhibitors has been a breakthrough in the development of cancer immunotherapy driver mutations in non-small-cell lung cancer [2]. The most significant challenge of molecularly targeted testing is obtaining a proper amount of tumor tissue. In many cases, the amount of FFPE (formalin-fixed, paraffin-embedded) tumor tissue obtained after a pathologist’s analysis is insufficient for molecular testing. Consequently, an additional biopsy (surgery) must be performed, which is an invasive procedure with associated risks.

Cell-free DNA (cfDNA) in plasma could serve as a substitute for formalin-fixed, paraffin-embedded, or fresh-frozen tumor tissues, as it can be obtained multiple times with low risk to patients. However, an important consideration is that tissue samples only represent a fraction of the tumor. A single section of the tumor typically does not capture the overall heterogeneity of the tumor. Tumor-specific genomics identified in cfDNA from patient blood samples can complement biopsies for real-time molecular monitoring of treatment, recurrence detection, and resistance tracking, particularly when tumor tissue is unavailable or insufficient for testing. Next-generation sequencing (NGS) has been successfully applied for blood-based genomic profiling.

Nevertheless, the utility of cfDNA as an informative tool in target mutation detection remains questionable, primarily due to complications in cfDNA extraction and the typically low concentration of cfDNA in blood.

Molecular genetic testing must be conducted promptly, as the selection of personalized treatment for lung cancer patients is time-sensitive. Various molecular testing methods are available, including qPCR, dPCR, and NGS. NGS, in particular, can identify a wide range of driver gene variations, including therapeutic target genes and acquired drug-resistant genes, simultaneously, and has seen increasing application in clinical practice [3].

In this study, our main objective was to compare lung cancer mutations in plasma and tumor tissue samples with the Ion AmpliSeq Colon and Lung Cancer Research Panel (Ion Torrent PGM platforms) method, aiming to assess whether plasma samples can replace tumor tissue samples for routine genetic testing.

## 2. Materials and Methods

### 2.1. Patients

The study has been approved by the Vilnius Regional Biomedical Research Ethics Committee (Vilnius, Lithuania). All participants in the study signed informed consent to participate before study-specific procedures started. Tumor tissue samples were collected at the National Cancer Institute (Vilnius, Lithuania) and Vilnius University Hospital Santaros Klinikos (Vilnius, Lithuania), NGS was performed at Klaipeda University Hospital (Klaipeda, Lithuania) (No. 158200-13-688-219).

All samples were obtained before treatment. From each woman, two samples were obtained: a lung tumor and plasma. Of the 51 Lithuanian females with NSCLC diagnosis, 40 cases (78%) were predominantly diagnosed with adenocarcinoma and 9 (18%) with squamous cell carcinoma. It is important to note that 21 (41%) of the total patients were smokers (Table 1).

### 2.2. DNA Preparation

Two types of samples were collected from each patient: tumor tissue (fresh or formalin-fixed paraffin embedded (FFPE)) and EDTA plasma. DNA was extracted from fresh tissue using the QIAsymphony DSP Virus/Pathogen Midi Kit (Qiagen, Hilden, Germany), from FFPE using the QIAamp DNA FFPE Tissure Kit (Qiagen, Hilden, Germany), and cfDNA was extracted from plasma using the QIAamp Circulating Nucleic Acid Kit (Qiagen, Hilden, Germany) following the manufacturers’ instructions. DNA was quantified with a spectrophotometer (Cary 60 UV-Vis, Agilent Technologies, Santa Clara, California, US), and cfDNA was quantified with Therascreen EGFR Plasma RGQ PCR Kit (Qiagen, Hilden, Germany).

### 2.3. Library Preparation and Sequencing

Library preparation and sequencing were performed using the Ion AmpliSeq Colon and Lung Cancer Research Panel (Ion Torrent PGM platform).

Libraries were amplified using the Ion AmpliSeq Colon and Lung Cancer Research Panel (ion torrent by Life Technologies, Carlsbad, California, US), which analyzes 92 amplicons in hotspots and target regions of 22 oncogenes (*KRAS, EGFR, BRAF, PIK3CA, AKT1, ERBB2, PTEN, NRAS, STK11, MAP2K1, ALK, DDR2, CTNNB1, MET, TP53, SMAD4, FBX7, FGFR3, NOTCH1, ERBB4, EGFR1, FGFR2*) with a coverage of over 500 mutations involved in colon and lung cancers. 10ng DNA and cfDNA were amplified using the Ion AmpliSeq Library Kit 2.0 (ion torrent by Life Technologies, California, US) following the manufacturers’ instructions. The library concentration was checked with Ion Library TaqMan Quantitation Kit (IonTorrent by Life Technologies, California, US). Each library was diluted to 100 pM, amplified with emulsion PCR, and then enriched using the Ion PGM 200 Sequencing Kit (ion torrent by Life Technologies, California, US). Sequencing was accomplished on the Ion PGM (ion torrent by life technologies, California, US) with the Ion PGM 200 Sequencing Kit (ion torrent by Life Technologies, California, US) loading libraries into a 316 chip following the manufacturers’ instructions.

### 2.4. Data Analysis

Bioinformatics conducted analysis to compare results between plasma and tumor; the minimum coverage for variant calling was set at a 100-read depth threshold and VAF (variant allele frequency) ≥ 1% for plasma and VAF ≥ 2% for FFPE for data generated by either sample. Based on noise estimation, a threshold was set for variant calling with a minimum requirement of 5 variant reads for both types of samples.

## 3. Patient Group

A group of 51 females with a diagnosis of NSCLC (clinical data, pathology diagnosis, tumor sample, and EDTA plasma).

**Figure 1:**
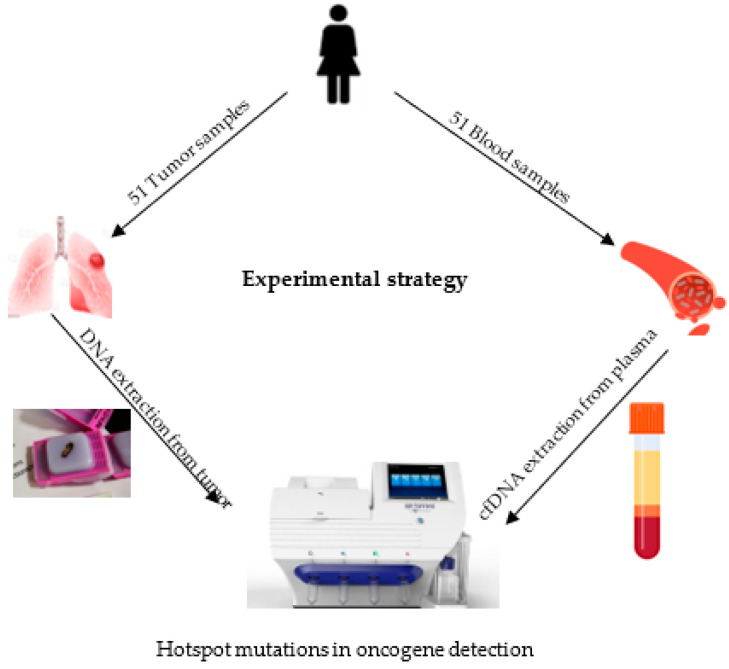
Experimental strategy.

DNA was extracted from 51 patients’ matched plasma and tissue samples. An experimental strategy is outlined in Figure 1.

## 4. Results

Concordance analysis was performed using VCF files from both plasma and tumor sequencing resultants. All samples were subjected to the same bioinformatics analysis. All variants were filtered according to read depth and limit of detection (LOD), as described above.

**Figure 2:**
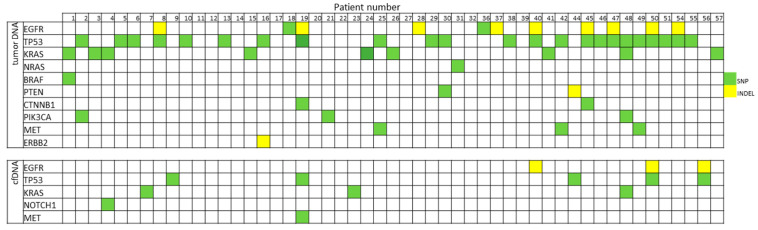
Gene mutations in tumor and plasma samples.

Target mutations were identified in 38 out of 51 patients (74.5%) in tumor tissue samples, whereas in plasma samples, target mutations were identified in only 10 patients’ samples (19.6%) (Figure 2).

**Figure 3:**
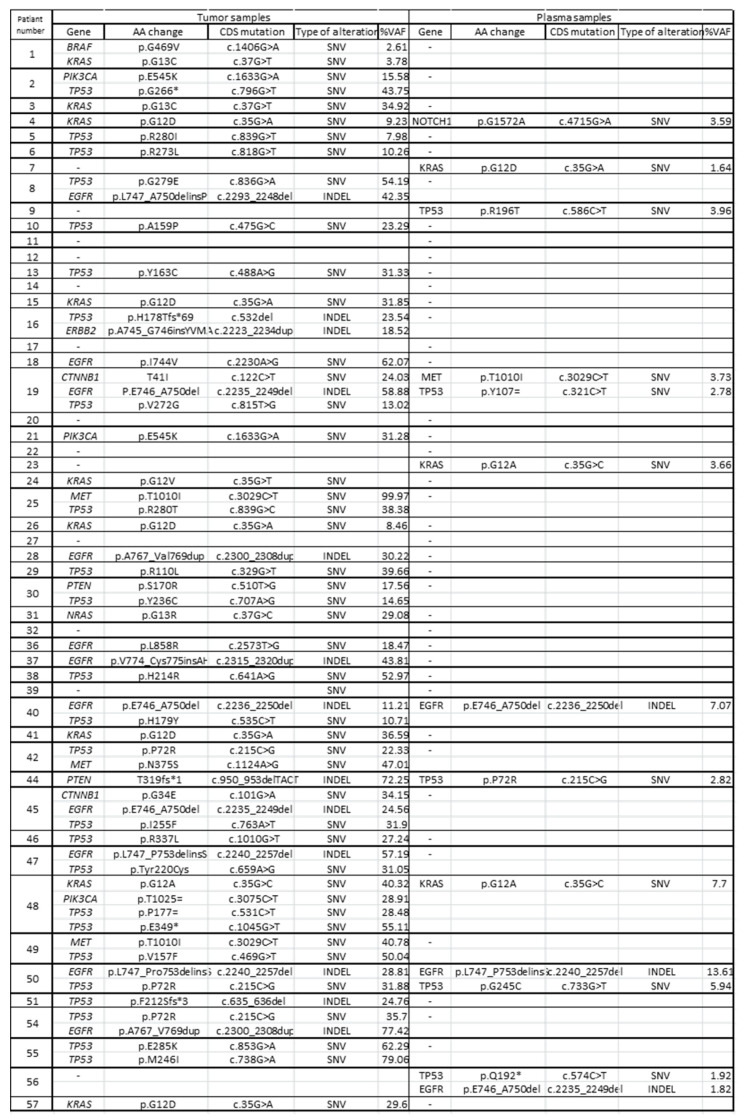
Gene mutation comparison in tumor and plasma samples. * means STOP codon.

Three mutations were detected in both tumor and plasma samples for the same female: *EGFR* p.E746_A750del (sample number 40), *KRAS* p.G12A (sample number 48), and *EGFR* p.L747_P753delins (sample number 50). In four female samples, mutations were detected only in plasma samples (no mutation detection in tissue samples). In 32 female samples, mutations were detected only in tumor tissue samples (no mutation detection in plasma samples) (Figure 3).

**Figure 4:**
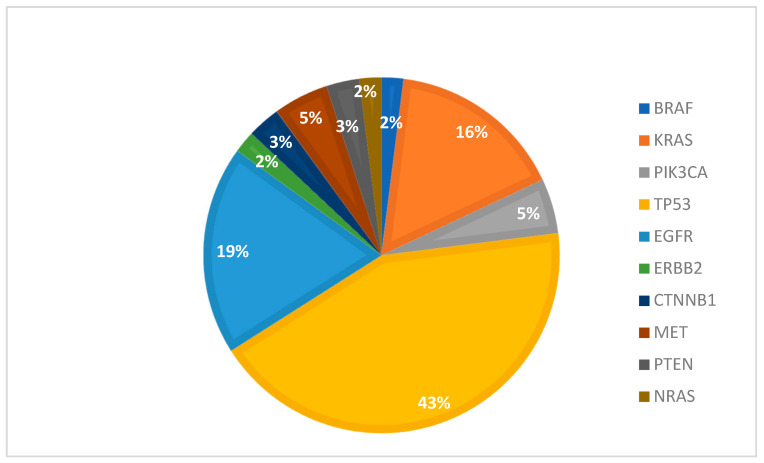
Percentage of gene mutations in tissue samples.

The most frequent mutations identified in tissue samples reside within the *TP53* (43.1%), *EGFR* (18.96%), *KRAS* (15.52%), and *MET* (5.18%) genes (Figure 4).

**Figure 5:**
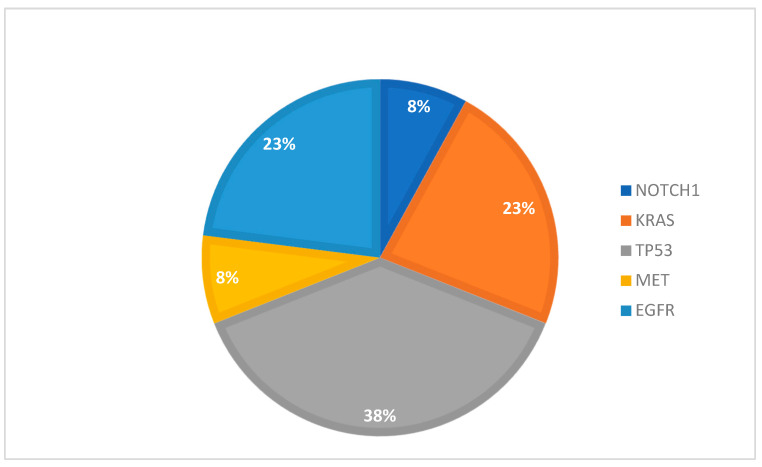
Percentage of gene mutation in plasma samples.

The most frequent mutations identified in plasma samples reside within the *TP53* (38.46%), *EGFR* (23.08%), and *KRAS* (23.08%) genes (Figure 5).

One of the main objectives of this study was to compare mutations in plasma and tumor samples, but the results did not reveal a significant correlation between them. However, it is worth noting that there was only ≤500 μL of plasma available for cfDNA extraction, which likely had a significant impact on our results. Insufficient cfDNA material may not provide accurate comparison results between plasma and tissue samples. Consequently, this renders an impartial comparison between tissue and plasma patient-matched samples very challenging.

A low-complexity cfDNA sample increases the probability of missing mutations identified in tissue. Furthermore, if these patients were diagnosed with early stage NSCLC, it is reasonable to expect that a small tumor will not release large amounts of ctDNA into the circulation. Previous studies have demonstrated a correlation between cfDNA levels and disease stage [4]. Another explanation lies in intra- and inter-tumor heterogeneity. A small section from a tissue biopsy may fail to capture the molecular heterogeneity of the tumor. Conversely, a cell-free DNA profile can not only capture the heterogeneity of the primary tumor but also provide insights into the metastatic tumor profile [5].

## 5. Discussion

Despite the small sample size in our study, gene mutations were detected in the plasma samples of ten women. Among these, three women were diagnosed with early stage non-small-cell lung cancer (stages I and II). For these patients, the following mutations were detected: a deletion in exon 19 of the *EGFR* gene and single nucleotide polymorphisms in the *TP53* and *MET* genes. All other women were diagnosed with stage III or IV lung cancer. This suggests that the later stages of cancer contribute more cfDNA in plasma, thereby making extraction less complicated. The deletion in exon 19 of the *EGFR* gene is one of the most detected mutations in lung cancer patients [6]. Moreover, this mutation is easily detected in all samples (plasma, FFPE, and fresh-frozen tissue) and using all detection methods (quantitative PCR (qPCR), digital PCR (dPCR), and NGS).

During our study, the results showed that among all detected mutations in the tissue samples, *TP53* gene mutations were highly recurrent (43.1%). It is noteworthy that cancer patients typically possess some form of mutation in the *TP53* gene. Even though *EGFR*, *KRAS*, and *BRAF* gene mutations are the most characteristic in lung cancer patients, *TP53* gene mutations are frequently detected as well, even in cases where no mutations are found in the *EGFR*, *KRAS*, and *BRAF* genes. Consequently, detecting *TP53* gene mutations in plasma samples could be utilized not only for clinical cancer diagnostics but also for prophylactic population screening [7].

It is important to note that a limiting factor in our study was the insufficient volume of plasma, as evident in the comparison of plasma and FFPE mutation spectra. Additionally, the quality of the cfDNA and the reliability of the cfDNA detection kit can also be limiting factors. Extracting nucleic acid from FFPE tissues yields highly variable results in terms of quantity and quality [8]. Therefore, DNA detection methods should adapt to these variations, such as using shorter, higher-concentration primers. However, cfDNA variability and quality may be worse than FFPE DNA quality, necessitating careful consideration when designing detection kits.

## 6. Conclusions

Theoretically, cfDNA from plasma could be a superior tool for lung cancer diagnostics and the detection of early cancer stages. Therefore, further improvements in cfDNA extraction and detection methods from plasma are needed, including the development of superior detection kits and analysis tools.

In patients diagnosed with NSCLC, *EGFR* gene mutations are the most common. These mutations lead to activation of the tyrosine kinase domain and are associated with sensitivity to targeted therapy with small-molecule tyrosine kinase inhibitors (TKIs) [9]. Therefore, mutation detection for disease monitoring or progression should ideally be conducted using cheaper, quicker, and more sensitive screening methods such as qPCR or dPCR.

Primer or metastatic tumor genomic profile detection with NGS should primarily utilize FFPE tissue, or cfDNA could serve as an alternative tool when the quality of available tumor biopsy and/or cytology material is inadequate for the necessary molecular testing.

## Figures and Tables

**Table 1:** Patient characteristics.

Characteristic	*n* (%)
Age (years)	
Mean (SD)	62.65 (9.85)
Median (range)	64 (40–81)
Non-small-cell lung cancer pathological diagnosis	51 (100%)
Adenocarcinoma	40 (78%)
Squamous cell carcinoma	9 (18%)
Large-cell carcinoma	2 (4%)
Tumor stage	
I	2 (3.9%)
IA	2 (3.9%)
IB	10 (19.6%)
IIA	6 (11.8%)
IIB	5 (9.8%)
IIIA	11 (21.6%)
IIIB	7 (13.7%)
IV	8 (15.7%)
Smoking status	
Non-smoking	29 (57%)
Smoking	21 (41%)
Unknown	1 (2%)
